# Efficacy of tart cherry juice in reducing muscle pain during running: a randomized controlled trial

**DOI:** 10.1186/1550-2783-7-17

**Published:** 2010-05-07

**Authors:** Kerry S Kuehl, Erica T Perrier, Diane L Elliot, James C Chesnutt

**Affiliations:** 1 Department of Medicine, Oregon Health & Science University, Portland, OR, USA, 97239; 2 Department of Orthopedics, Oregon Health & Science University, Portland, OR, USA, 97239

## Abstract

**Background:**

Long distance running causes acute muscle damage resulting in inflammation and decreased force production. Endurance athletes use NSAIDs during competition to prevent or reduce pain, which carries the risk of adverse effects. Tart cherries, rich in antioxidant and anti-inflammatory properties, may have a protective effect to reduce muscle damage and pain during strenuous exercise. This study aimed to assess the effects of tart cherry juice as compared to a placebo cherry drink on pain among runners in a long distance relay race.

**Methods:**

The design was a randomized, double blind, placebo controlled trial. Fifty-four healthy runners (36 male, 18 female; 35.8 ± 9.6 yrs) ran an average of 26.3 ± 2.5 km over a 24 hour period. Participants ingested 355 mL bottles of tart cherry juice or placebo cherry drink twice daily for 7 days prior to the event and on the day of the race. Participants assessed level of pain on a standard 100 mm Visual Analog Scale (VAS) at baseline, before the race, and after the race.

**Results:**

While both groups reported increased pain after the race, the cherry juice group reported a significantly smaller increase in pain (12 ± 18 mm) compared to the placebo group (37 ± 20 mm) (p < .001). Participants in the cherry juice group were more willing to use the drink in the future (p < 0.001) and reported higher satisfaction with the pain reduction they attributed to the drink (p < 0.001).

**Conclusions:**

Ingesting tart cherry juice for 7 days prior to and during a strenuous running event can minimize post-run muscle pain.

## Introduction

Long distance running is known to cause acute muscle damage resulting in acute inflammation [1] and decreased force production [2] that can last up to 1 week post-exercise [3]. One proposed mechanism for this acute response to distance running is that extensive myofibril disruption triggers a local inflammatory response, exacerbating muscle damage [4-9]. Leukotrienes then increase vascular permeability, attracting neutrophils to the injury site, resulting in free radical production [10]. Among endurance athletes, NSAIDs are used during competition to prevent or reduce pain during a race [11]. There are, however, known adverse effects associated with the use of traditional oral NSAIDs [12], including gastrointestinal, renal, and cardiovascular adverse events. Tart cherries are considered a good source of phenolic compounds with high levels of antioxidant and anti-inflammatory activity [13,14]. Considering the natural anti-inflammatory and antioxidant capacity of tart cherries, it is plausible that cherry consumption before and during strenuous exercise may have a protective effect to reduce muscle damage and pain.

Consumption of approximately 45 cherries per day has been shown to reduce circulating concentrations of inflammatory markers in healthy men and women [15,16]. Moreover, a recent study of healthy, exercise-naïve individuals demonstrated efficacy for cherry juice in decreasing symptoms and strength loss following eccentric exercise induced muscle damage. Most notably, there was a preservation of muscle function attributable to the cherry juice [15]. The specific anti-inflammatory mechanism by which cherry juice supplementation may lessen exercise-induced muscle damage is not well understood [16]. However, it is possible that the anti-inflammatory and/or the antioxidant effects of cherry juice may mediate this secondary response and avoid the proliferation of myofibrillar disruption [17]. While there are no studies directly measuring neutrophil and monocyte activation after exercise, this mechanism may represent a potential explanation for the reduction in inflammation and strength losses associated with tart cherry consumption.

The Oregon Hood to Coast relay race presented a unique opportunity to examine the effects of tart cherry juice supplementation on acute muscle damage caused by repeated bouts of running. Covering 315 km from Mt. Hood to the Oregon coast, the race involves relay teams of 12 runners who complete 3 race segments each (individual total running distance: 22.5 to 31.4 km). Crossing two mountain ranges, the hilly course provides ample opportunity for eccentric muscle damage, with individual running segments descending up to 609 m or ascending up to 200 m. The purpose of this study was to assess the effects of tart cherry juice, compared to a placebo cherry drink, on muscle pain among Hood to Coast runners.

## Methods

### Subjects

Fifty-four healthy runners participating in the Hood to Coast relay (36 male, 18 female; 35.8 ± 9.6 yrs) volunteered to participate. The study was approved by the university's Institutional Review Board and by the Hood to Coast race director, and all participants gave written, informed consent. Inclusion criteria included an ability and willingness to abstain from anti-inflammatory or pain-relieving drugs, and willingness to refrain from seeking any other treatment for symptoms of muscle damage until the completion of the study. Exclusion criteria included recent use of other pain management methods (including acupuncture, transcutaneous electrical nerve stimulation, topical medications/anesthetics, muscle relaxants, injections, or systemic steroids). Women capable of becoming pregnant completed a pregnancy test to rule out pregnancy prior to participation.

### Beverage Preparation

#### Cherry Juice

The cherry juice was prepared by mixing freshly prepared tart cherry juice with commercially available apple juice in a proprietary ratio (Cherrish Inc., Seattle, WA, USA). Frozen tart cultivar Montmorency cherries were used to prepare the cherry juice following standard procedures that simulate industrial processing. The blended juice was pasteurized by heating it to 85°C, hot packed into 10.5 oz plastic bottles with a three minute hold time to achieve commercial sterility, and then forced cooled in a water bath. One 10.5 oz bottle of the juice provided at least 600 mg phenolic compounds, expressed as gallic acid equivalents by the method of Singleton and Rossi [18], and at least 40 mg anthocyanins, calculated as cyanidin-3-glucoside equivalents by the pH differential method described by Giusti and Wrolstad [19]. Each bottle contained the equivalent of 45-50 cherries.

#### Placebo

The placebo was prepared by mixing unsweetened fruit punch soft drink mix (Kraft Corporation, Ryebrook, New York, USA; ingredients listed: citric acid, salt, calcium phosphate, red 40, artificial flavor, ascorbic acid, blue 1) with water in the proportion recommended by the manufacturer (about 2 g/l). Sugar was added to match the concentration of soluble solids in the cherry juice blend to a final concentration of 13 Brix (total percentage soluble solids by weight). The flavored beverage was then pasteurized and bottled following the procedure used for the juice.

### Experimental Design

**Figure 1:**
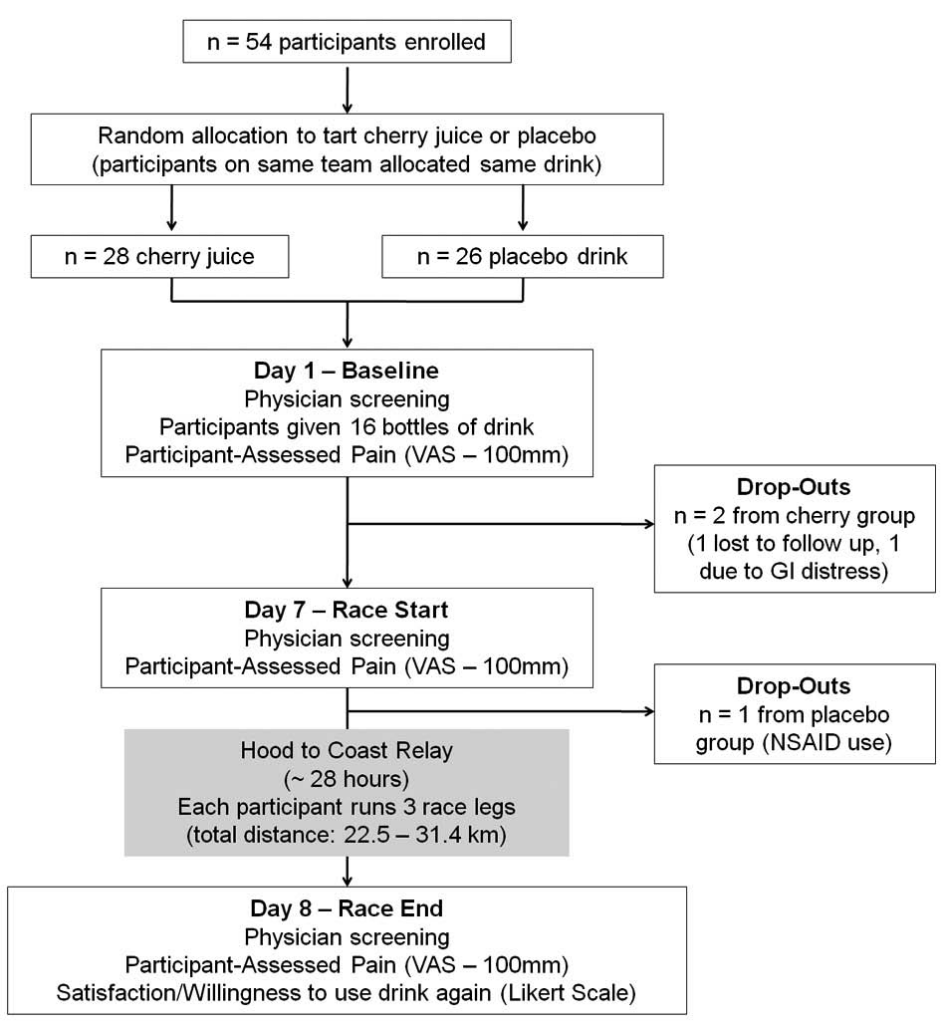
**Consort diagram of enrolled participants**.

The design was a randomized, placebo-controlled, double-blind trial among 54 runners participating in the Hood to Coast relay race (Figure 1). Each participant completed 3 running segments during the race, with individual segment distances ranging from 5.6 to 12.4 km and an average total running distance of 26.3 ± 2.5 km. Participants running on the same relay team were assigned to the same drink condition (n = 28 cherry; n = 26 placebo) in order to avoid participants inadvertently switching drinks during the study. Participants completed 3 data collection sessions: Day 1 - Baseline (7 days prior to race), Day 7 - Race Start, and Day 8 - Race End. At Baseline, participants were given 16-355 mL bottles of the drink (cherry juice or placebo) with instructions to consume two bottles daily prior to the race (14 bottles over 7 days), and two bottles during the race (total consumption: 16 bottles). Baseline data collection also included a health screening by a physician blinded to the participant's drink condition. Participants assessed their pain intensity during each visit on a standard 100 mm Visual Analog Scale (VAS), with 0 mm indicating 'no pain', and 100 mm indicating 'most severe pain'. The VAS has excellent reliability for acute pain [20] as well as well-defined thresholds for meaningful change in pain intensity [21]. After finishing the race (Day 8), participants completed the VAS pain scale and a short questionnaire reporting their level of satisfaction with the pain relief they attributed to the drink.

### Statistical Analysis

Outcome variables were: participants' assessment of pain (VAS), level of satisfaction with the drink, and willingness to use the drink in the future. VAS pain scores were analyzed using [3 (time) × 2 (drink)] mixed-effects regression (SPSS version 16 for Windows, Chicago, IL). Participant satisfaction and participant willingness to use the drink again were analyzed using independent samples t-tests. Level of significance was set at α = 0.05.

## Results

### Baseline Participant Demographics

Of the 54 participants enrolled, 28 were assigned cherry juice and 26 were assigned the placebo drink (Table 1). A total of 3 participants (2 cherry, 1 placebo) withdrew prior to competing the study (1 was lost to follow-up; 1 reported that the drink caused GI distress; 1 took NSAIDs during study period). Despite the fact that participants were randomized into treatment groups, the cherry group reported significantly higher pain scores than the placebo group on Day 1 (F(1,49) = 8.00; p < 0.01).

**Table 1:** Participant baseline demographics

	Placebo	Cherry
N	25	26
Age	32.2 ± 9.8	38.2 ± 8.5
Male/Female	15/10	19/7
Baseline VAS (mm)*	6.1 ± 7.9	16.1 ± 15.9

* Baseline VAS significantly different between groups (p < 0.01)

### Pain (VAS) at Race Start and Race End

Mixed-effects regression revealed significant main effects of drink (F(1,49) = 11.50; p < 0.01), time (F(1,49) = 85.51, p < 0.001) as well as an interaction between drink and time (F(1,49) = 22.64, p < 0.001). At Race Start, there were no differences in mean VAS score between the cherry and placebo groups (p = 0.38). After completing the race, participants in both groups reported more pain; however, the increase in pain was significantly smaller in the cherry juice group compared with the placebo group (p < 0.001) (Table 2).

**Table 2:** Mean pain scores (VAS) at 3 time points (baseline, race start, race end)

	Day 1 (Baseline)	Day 7 (Race Start)	Day 8 (Race End)
Placebo	6.1 ± 7.9	8.0 ± 9.6	45.3 ± 20.5
Cherry	16.1 ± 15.9*	10.6 ± 11.8	22.6 ± 12.6**

Between groups: * p < 0.05; ** p < 0.001

### Participant Satisfaction

Participants in the cherry juice group reported higher willingness to use the drink again (p < 0.001), higher overall satisfaction with the drink (p < 0.001), and higher satisfaction in the pain reduction they attributed to the drink (p < 0.001) (Table 3).

**Table 3:** Participant satisfaction with drink

Measure		Mean Score	*p*
Willingness to use drink in future (1 = very unwilling; 10 = very willing)	Placebo	5.0 ± 2.5	*< 0.001*
	Cherry	8.3 ± 1.3	
			
Drink Satisfaction - Pain Relief (1 = very satisfied; 5 = very dissatisfied)	Placebo	3.6 ± 0.9	*< 0.001*
	Cherry	2.2 ± 0.6	
			
Drink Satisfaction - Overall (1 = very satisfied; 5 = very dissatisfied)	Placebo	3.3 ± 0.8	*< 0.001*
	Cherry	2.1 ± 0.5	

## Discussion

It is well-documented that running for distances in excess of typical training distances causes acute muscle injury, and that eccentric muscle actions, such as downhill running, exacerbate injury and soreness [22]. The Hood to Coast relay requires participants to run three separate race segments over an approximately 24 hour period, including segments that ascend or descend steep terrain. It is expected, therefore, that Hood to Coast runners will experience inflammation and pain during the strenuous race. In our study, runners in both groups reported more pain upon completion of the race. However, participants who drank the tart cherry juice twice daily for one week prior to and the day of the race reported a significantly smaller increase in pain after the race (mean post-race increase of 12 mm in the cherry juice group, compared with a 37 mm increase in the placebo group). The relative post-race reduction in pain in the cherry group (25 mm lower VAS than placebo) suggests that tart cherry juice provided a protective benefit against the acute muscle pain caused by distance running.

Pain associated with acute muscle injury is most likely due to oxidative tissue damage which leads to an inflammatory response, causing further production of free radicals and augmenting secondary muscle soreness [23-25]. Because of that pathogenesis, nutritional antioxidants have been proposed as a means of mitigating muscle soreness and strength loss caused by damaging exercise [15]. Tart cherries contain flavinoids and anthocyanins, with high antioxidant and anti-inflammatory properties [13,14]. Consumption of about 45 cherries a day has been shown to reduce circulating inflammatory markers in healthy men and women [16]. Moreover, Kelley et al. reported that serum inflammatory markers including C-reactive protein (CRP) decreased by 25% after 28 days of consuming Bing sweet cherries [26]. Additionally, when studied in healthy young adults, consumption of cherry juice equivalent to 100-120 cherries daily reduced strength loss and pain associated with exercise-induced delayed-onset muscle soreness (DOMS) [15]. In our study, participants consumed two 355 mL bottles of tart cherry juice daily, (~90 to 100 cherries) for just seven days prior to and on the day of the race. The attenuated pain in the cherry juice group suggests that even short term (~1 week) supplementation with tart cherry juice is effective at reducing the acute pain caused by repeated bouts of distance running. Our results are similar to those reported by Howatson et al. [27], in which runners who consumed tart cherry juice for 5 days prior to and 48 hours after a marathon showed faster recovery of muscle strength as well as reduced inflammation.

Due to methodological limitations, our results should be interpreted with caution. One limitation to the study was the subjective of assessment of pain by participants. However, the VAS is commonly used to determine acute levels of pain and has consistent and well-defined clinically meaningful thresholds [21,28]. A second limitation is the possibility of cross contamination of the intervention and placebo drinks, as participants may have potentially switched drinks to compare flavor and effects. This limitation was addressed by assigning participants on the same relay team to the same beverage condition.

## Conclusions

In conclusion, tart cherries have high levels of antioxidant and anti-inflammatory compounds, and are promoted in lay publications as beneficial for those with arthritis, muscle pain, and fibromyalgia. The nutraceutical industry is experiencing exponential growth and defining for whom these products might be beneficial is an important task. The present study suggests that the administration of tart cherry juice for eight days reduced symptoms of exercise-induced muscle pain among runners participating in a vigorous endurance event. Further research is needed to examine serum biomarkers and the potential explanation for the reduction in pain and inflammation associated with tart cherry consumption.

## Competing interests

The authors declare that they have no competing interests.

## Authors' contributions

KK, DE, and JC conceived of the study, participated in its design and coordination and helped to draft the manuscript. EP carried out the analysis and interpretation of the data, and drafted the manuscript. All authors read and approved the final manuscript.
